# Teaching in the Suburbs: Participatory Action Research Against Educational Wastage

**DOI:** 10.3389/fpsyg.2019.02308

**Published:** 2019-10-18

**Authors:** Santa Parrello, Ilaria Iorio, Filomena Carillo, Cesare Moreno

**Affiliations:** ^1^Department of Humanities, University of Naples Federico II, Naples, Italy; ^2^Non-profit Association Maestri di Strada, Naples, Italy

**Keywords:** teacher participatory action research, prevention of educational wastage, suburban schools, teachers’ resilience, teachers’ self-reflection, ALCESTE text analysis

## Abstract

If teaching is a stressful job, it can be even more so in schools in disadvantaged areas, such as the metropolitan suburbs, where the rates of educational wastage are high. Here, teachers often feel ineffective: as a result, there is a reduced sense of well-being at work, which triggers a negative cycle that damages their educational performance. From the literature, it is known that teachers need social support, which has a positive effect on well-being and resilience. For these reasons, the Association “Maestri di Strada” (MdS) has chosen to offer teachers professional social support and to actively involve them through Teacher Participatory Action Research (T-PAR): the “Crossing Educational Boundaries” project. These are the research questions that gave life to the project: do the teachers have resources to analyze the problematic situations they are immersed in and to build improvement strategies? Would a professional social support reinforce their resilience? The objective was the following: to actively engage the teachers in order to generate hypotheses concerning the causes of educational wastage in their schools, and to work with them to plan new methods to lessen the problem. The project was carried out in 12 suburban secondary schools in six Italian cities. This paper illustrates the activities of three cities. All phases of the T-PAR were completed. The teachers organized discussion groups and started workshops in the classes considered at risk. The activities were subject to non-participant observation, and the observation reports underwent semantic-structural analysis. Four clusters emerged the analysis. The results show that the teachers are aware of the importance of a good educational relationship as a way to oppose educational wastage, and, at the same time, they are aware of the difficulties of building it, which they attribute to the mistrust and passiveness of the pupils, and to the demands of the institution. The moments of discouragement shown by the teachers, and their strong emotional engagement in the pupils’ difficulties are significant. At the end of the project, a small group of teachers planned and implemented a reflective space in some of the schools.

## Introduction

Teaching is a stressful and tiring job for a complex variety of reasons linked to multiple factors, including: the nature of the work, which implies a high degree of relationality; personal characteristics, on both a social and emotional level; and school organization, which is influenced by the historical and geographical context (Kyriacou, 2001; Hastings and Bham, 2003; Hakanen et al., 2006; Bizumic et al., 2009; Chang, 2009; Day and Gu, 2009; Zurlo et al., 2016; Steffens et al., 2017; Capone et al., 2019; Parrello et al., 2019a). To this end, an extensive array of literature testifies to the importance of cultivating well-being and resilience in teachers, and identifies protection factors and intervention strategies.

In particular, resilience in teachers is defined as a quality that enables teachers to maintain their commitment to teaching and to their teaching practices, despite challenging conditions and recurring setbacks (Brunetti, 2006). Resilient teachers have been described as those who have the capacity to thrive in difficult circumstances, are skilled in behavior management, are able to empathize with difficult students as well as restrain negative emotions and focus on the positive, and who experience a sense of pride, fulfilment, and increased commitment to their school and profession (Howard and Johnson, 2004). Resilience involves the capacity of an individual teacher to harness personal and contextual resources to navigate through educational challenges and to facilitate the outcome of professional engagement, growth, commitment, enthusiasm, satisfaction, and wellbeing (Beltman, 2015). In recent years, researchers have begun to conceptualize resilience from a social-ecological perspective, wherein resilience is defined as a set of behaviors over time that reflect the interactions between individuals and their environments, in particular the opportunities for personal growth that are available and accessible (Ungar, 2012). Some authors speak of relational resilience (Le Cornu, 2013; Gu, 2014): in particular, reciprocal and mutually supportive personal, professional and peer relationships are important in this process (Sammons et al., 2007). The outcome is that teachers maintain job satisfaction and commitment to their profession (Brunetti, 2006). Many authors do not explicitly examine resilience, but do address the question of what sustains teachers and what enables them to thrive rather than just survive. These papers could be grouped into three categories, each with a different focus: those emphasizing individual factors; contextual factors; and individual perceptions of, and responses to, the specific contexts of teacher work (Beltman et al., 2011).

The urban suburbs are among the contexts considered at risk. If teaching is always difficult, it can be even more so in schools in these disadvantaged areas. In suburban schools, teachers have to deal with adolescents of lower socio-economic status who often experience distress in their personal lives as well as at school (Sommantico et al., 2015; Pellerone et al., 2018; Lavy and Ayuob, 2019), and who have an internalized marginality which becomes a learned sense of powerlessness (David, 2014). Teaching in these environments therefore means: interacting and connecting daily with young people who are struggling with problems and pain, as well as working to decrease educational wastage. In these environments, we find both material and educational poverty: the material poverty of one generation often causes the deprivation of educational opportunities for the next (Jones, 2003; Lott, 2012; OCSE, 2015). In order to break this vicious circle, schools in deprived areas will need more funds and investments. In Italy, on the contrary, schools populated by disadvantaged students tend to have fewer resources (OCSE-PISA, 2012). Consequently, we can identify a socio-economic map showing the distribution of young people with a serious deficiency in the fundamental skills needed to grow and work in the world, to which we can also add a map of early school leavers.

According to UNESCO (1972), *educational wastage* is the combination of repeated grades and early school leaving. In Italy, this phenomenon reaches alarming levels: in 2014, the “Educational Wastage” Dossier complied by Ministry of Education, University and Research [MIUR] (2014) observed the presence of almost three million young people who did not complete upper secondary education. In 2015, Italy reduced the rate of early school leavers, although the percentage was still higher than the EU average (Istat, 2015). Over time, experts have highlighted different causes for educational wastage. In the ‘60s and ‘70s, they focused especially on its *socio-economic factors*. In the ‘80s, they additionally considered some of the students’ *subjective dimensions*. Over the same period, researchers began studying the *relational dimension* concerning students and teachers: increased attention was paid to classroom management styles, to the active or passive role of the students, and to the language used in the educational environment (Batini and Bartolucci, 2016). If we take this relational standpoint, we cannot speak of “wastage” in the singular, but rather of “wastages,” plural (Perone, 2006), or of a wasteful system: the situation does not concern only those who leave school entirely, but also those who attend without learning, as in the case of in-school drop-outs (Solomon, 1989). The waste is not limited to the potential of students: it also extends to the work of teachers and to school equipment (Brimer and Pauli, 1971). Contributions from psychoanalysis, which study affective/emotional dynamics, processes of symbolization in inter-generational relationships, and relationships with institutions, belong to the same perspective (Blandino and Granieri, 1995). Depending on the different theoretical approaches, a succession of interventions aimed at *strengthening teaching* have been employed over the years, for example through the repetition and simplification of the material being taught, as well as interventions aimed at *motivating* and *re-motivating* students. There have also been interventions aimed at improving the *quality of the educational relationship*, which encompassed a variety of the teacher’s relational skills, e.g., their emotional skills (Nouwen et al., 2016; Molloy Elreda et al., 2019). Fewer studies have focused on the importance of developing resilience in teachers if they are, in turn, to foster this trait in students (Bobek, 2002; Henderson and Milstein, 2003), even though resilience as a personal characteristic has been studied extensively in at-risk students (McMillan and Reed, 1994; Johnson, 1997; Aronson, 2001). Day and Gu (2014) have cogently argued that “efforts to increase the quality of teaching and raise standards of learning and achievement for all pupils must focus on efforts to build, sustain and renew teacher resilience, and that these efforts must take place in initial teacher training” (p. 22).

### The Work of the Association MdS to Contain Educational Wastage

The non-profit Association Maestri di Strada (MdS) was born in Naples in 2003 with the Chance Project, a second chance school for drop-outs, recognized as an “activity of excellence” by the European Union: MdS carries out complex socio-educational interventions inside and outside suburban schools, in order to prevent educational wastage and to promote social inclusion (De Rosa et al., 2017). In the last 10 years, in line with several European approaches to reduce early school leaving (Nouwen et al., 2016), MdS has chosen to work especially in schools in the eastern suburbs of Naples: they support teachers in the classroom and share different methods with them.

The environment of the eastern suburbs of Naples is characterized by social and economic marginality, unemployment, environmental neglect, a lack of public services, the widespread presence of organized crime, and educational wastage. In this context, teachers feel ineffective or powerless, and as if their job has lost meaning. It is not easy to work in these environments for MdS, either. The relationships with children, teenagers, families and teachers, all charged with problems and distress, are trying. It is just as stressful to connect with educational or political institutions, because it entails facing different forms of inflexibility. MdS takes care of the well-being of its partners and encourages their *professional reflection*, *communication and cooperation* through observations, narrations, and group meetings. *Observation* (McMahon and Farnfield, 2010) is employed both for on-field educational activities and for reflective group meetings. All partners are also asked to write *narrations* about their work throughout the year. The *multi-vision group* (Parrello et al., 2019b) is the method chosen to support professional reflection: it was inspired by the Balint group (Van Roy et al., 2015). The results of the Association’s efforts are encouraging: at the end of each school year, 90% of the kids they care for stay in school and succeed in moving on to the next school year (Parrello, 2018).

Spending considerable time inside of schools, MdS partners become witnesses of the hardships of many teachers and suggest activities to support their work. For these reasons, MdS has chosen to offer teachers professional social support, to support their resilience, and to actively engage them through T-PAR methodology.

From the literature, it is known that teachers need social support, both internal and external to the school system. In fact, good professional relationships have a positive effect on well-being, sense of engagement, empowerment, self-efficacy and the resilience of teachers in their work (Betoret, 2006; Halbesleben, 2006; Skaalvik and Skaalvik, 2009; Soini et al., 2010; Brouwers et al., 2011; Avanzi et al., 2018). The resilience of teachers is linked to the social support that they receive from colleagues, family and other professionals (Stanford, 2001), as well as to a “relevant, rigorous and responsive” education (Cefai and Cavioni, 2014, p. 144). The T-PAR methodology is a form of teachers’ education via Action Research.

Action Research (AR), as it is known, is an investigation model whose main aim is to improve the future skills and activities of the researcher, rather than produce theoretical knowledge (Lewin, 1948). In the ‘50s, Corey (1953) promoted AR in the United States in the field of education, gaining great cooperation from school districts and teachers: his method was later called *Cooperative Action Research*. At the end of the ‘60s, an international network of researchers created *Participatory Action Research* (PAR) in order to tackle various problems with *disadvantaged* members of society (Susman and Evered, 1978; Hall, 1992; Fals-Borda, 2001; Kidd and Kral, 2005; Arcidiacono et al., 2016). McTaggart (1997) explains that the dual objective of PAR is, on the one hand, to intervene in the situation being researched, and, on the other, to change the researchers themselves, activating in them a process of transformation that will make them agents of their own changes. From then on, PAR has been applied to many fields and disciplines, and it has assumed a much more critical position with respect to the broader field of AR, given its specific objective of dealing with power imbalances that generate social and personal distress (Arcidiacono et al., 2017; Stapleton, 2018). For this reason, the PAR method is thought to be particularly suited to the educational field (T-PAR), where power issues are a constant influence (Jacobs, 2016). Hooks (1984) discusses the power issue found in traditional educational environments: there are power discrepancies between teacher and pupil that start with teachers grading their pupils; however, this power can be used in non-coercive ways to improve the learning process.

Teachers often perceive a divide between theory and practice, between being able to think and having to do, whereas in PAR, knowledge is built by the people involved in the research process, in a non-hierarchical, democratic environment which ultimately constitutes “a social enterprise” (Savin-Baden and Wimpenny, 2007) that produces “a contextual knowledge” (Pine, 2009, p. 31). PAR can change both teachers and students, and also their mutual perception (Brydon-Miller and Maguire, 2009) and the emotions dominating the classroom (Hooks, 1994). Moreover, according to multiple authors, in-classroom research is in itself a way to promote self-reflection (Schön, 1983; Alber and Nelson, 2002), improving the teachers’ self-awareness, their control over their emotions and actions and over their own power, thereby reducing the pressure created by the context and finding resources where they previously only saw limits. More specifically, reflection – as part of the PAR cycle – is a meta-cognitive process that consists of exploring personal beliefs, thoughts and actions in a deliberate, autobiographical, and critical way (Marcosa et al., 2009). Thus, PAR is considered to be a powerful form of professional development for teachers (Johnson and Button, 2000), who are nevertheless usually reluctant to participate in action research: they do not comprehend how research could improve their work, because they lack the knowledge and training to see the connection between theory and practice (Bondy, 2001).

The PAR cycle is composed of the following phases: planning, action, reflection and evaluation. Some common characteristics of PARs are active participation, open-ended objectives and high levels of commitment (Greenwood et al., 1993; Morales, 2016). Multiple authors report the following various benefits of action research, which often go beyond the goal of the research project: improved teaching practice; enhanced collegiality; feelings of closeness to one another after working on a group research project; and becoming more reflective about the improvement of student performance (Glanz, 2003).

## Materials and Methods

In the academic year 2017–2018, MdS carried out the T-PAR project “Crossing Educational Boundaries”: the title is a reference to the importance of making education “cross the boundary” and leave the outskirts of society’s interests; it also alludes to the necessity of going beyond physical and mental limits, thus letting professionals, disciplines and methods meet in a free and creative way.

These are the *research questions* that gave life to the project: do the teachers have resources to analyze the problematic situations they are immersed in and to build improvement strategies? Would a professional social support reinforce their resilience?

The *objective* was the following: to actively engage the teachers in order to generate hypotheses concerning the causes of educational wastage in their schools, and to work with them to plan new methods to lessen the problem.

### Participants

The schools that participated are all located in the suburbs and have a high rate of educational wastage. 12 suburban schools participated, with about 200 teachers and 20 classes from different Italian cities: two metropolises (Rome, Naples), two medium-sized cities (Bologna, Florence), and two smaller provincial towns from the North and South (Rozzano, Sciacca). This paper will describe the first phase of the PAR project carried out in the first three participating schools – Rome, Bologna, Naples.

#### The School in Rome

Rome is a metropolis (population of city and provinces: almost four and a half million) in central Italy. The school that participated in the project is a Higher Technical Institute in a suburban area. The students are between 14 and 18 years old, and many do not obtain a high school diploma. Here, 15 teachers participated in the PAR.

#### The School in Bologna

Bologna is a medium-sized city (population of city and province: about 1 million) of Northern Italy. The school that participated in the project is a Lower Secondary School (students between 12 and 14 years old) located in a suburban area, where the teenagers attending are mostly children of immigrants. A few years ago, some Italian students also returned to the school, because the principal added a class with a curriculum using high-technology teaching. However, the result was a clear-cut separation between Italians and foreigners. Here, 14 teachers participated in the PAR.

#### The School in Naples

Naples is a metropolis (population of city and province: about 3 million) in Southern Italy. Its suburbs are known for the high rate of educational wastage, unemployment and organized crime. In this Lower Secondary School, teachers have been cooperating with MdS for some time, putting on workshops and accepting the help of its partners. This school’s biggest problem is educational wastage in its many forms. Here, 10 teachers participated in the PAR.

### Procedure

The project was presented to the school principals and teachers. MdS offered a preliminary time schedule, a team of the Association’s partners (pedagogues, psychologists, educators, workshop teachers, observers) and a “menu” of possible tools. The five phases of PAR have been planned and implemented, albeit in a specific way, in each school:

1. Planning: construction of a space – guided by a conductor of MdS – in which the teachers can meet to share experiences, think and plan freely.
2. Action: realization of educational actions (for example laboratories) guided by teachers with the support of MdS.
3. Reflection: construction of a space – guided by a conductor of MdS – to reflect together on the actions carried out.
4. Assessment: collection and analysis of materials useful for evaluating the path of PAR (for example observation reports); common discussion of the results.
5. New planning: formation of a group of teachers that, in collaboration with MdS, proposes prototypes of intervention.

In agreement with the teachers, all activities were observed by psychologists from the MdS Association, in the non-participant role (Ahola and Lucas, 1981). Observation is used regularly by researchers to collect data in classrooms (Kawulich, 2012). However, observing is not a natural ability, but rather the result of precise training: for this reason trained psychologists who belong to the association have been involved.

Observers were presented to teachers and students, explaining their role. They took notes during the observation. They had the task of observing freely, with particular regard to the activities and interactions that occurred in the setting: the non-verbal and verbal behaviors, and the conversations between participants.

However, the observer, whether he is aware of it or not, is a deformed and deforming mirror: some consider subjectivity as a risk to be avoided if possible because it is a source of error, while others see it as a resource, as a further component of cognition and therefore they consider subjectivity a main path to knowledge (McMahon and Farnfield, 2010). To limit the threshold of subjectivity inherent in observation, they were asked to use denotative and descriptive language, referring to precise (not generic) situations. They were also asked to include direct speech in the written notes. They were asked to insert their own comments and report them as such.

Observation reports were read out to research participants after the activities. They were then analyzed in a descriptive and content-oriented way in order to report processes and outcomes resulting from phase 1–3 in each school. Then, the reports related to phases 1–3 were analyzed in a lexical-oriented perspective in order to be used as an input for discussion in phase 4.

In particular, the corpus composed of all reports from the three schools was subjected to textual analysis by ALCESTE software (Analyse des Lexèmes Co-occurrents dans les Énoncés d’un TExte) (Reinert, 1990, 1993). The adoption of computerized textual analysis software enables researchers to overcome several limitations that are likely to occur in the manual coding of text: the time-consuming nature of manual coding, the potential for rater bias, the reliability and accuracy of coding as a result of researcher fatigue, and the practical difficulties of coding large data sets (Illia et al., 2012). It is noted that, on the other hand, this type of analysis reduces the complexity of the meaning making: the data obtained therefore only represent some aspects of this complexity. In particular, we chose the software ALCESTE (Analyse des Lexèmes Co-occurrents dans les Énoncés d’un TExte) (Reinert, 1990, 1993): it is an instrument of statistical analysis that explores the inner organization of a text through the concurrent presence or co-occurrence of several *content words*. The “positioning text analysis” makes it possible to make sense of a word based on its natural context. It is based on the fundamental assumption that “since the meaning of words is learned through reading and hearing them used in particular combinations, word co-occurrence is a suitable basis for representing meaning” (Ocasio and Joseph, 2005, p. 165). Discourse is conceived as a semantic space, and a word is considered based on the position it takes in this space. The use of ALCESTE is advisable for analyzing a set of data using a non-predefined dictionary which scans the entire text so as to include idiomatic language.

ALCESTE develops a spreadsheet with the list of words in the columns, and pieces of text or extracts in the rows (a typical extract – not necessarily corresponding to a set of sentences – is between 16 and 19 words long) and, based on this, produces a two-by-two matrix to identify classes of discourse which are very different. In technical terms this procedure is called “*hierarchical downward classification of words*.” The software compares how words co-occur or do not co-occur in each extract and develops a classification tree that is descendant, since the whole text is divided first into two large classes of discourse, each with the most differentiated use of words. Then, for each of these two parts, the software again divides the text into two further parts, which are differentiated, certainly, but less so than the first ones. The software continues this classification until the differences among classes of discourse become too small to be significant. At this third step, the researcher does not yet interpret the results. Instead, next, ALCESTE begins co-occurrence analysis by benchmarking different parts of the text. This benchmark is developed two times, which provides a measure of stability for the comparison (stability index). Thus, here one gets a measure of stability for the descending hierarchical classification. A good measure of stability is said to have been obtained when about 70%^1^ of the text is classified in the same way twice (Illia et al., 2012). Only in the second phase of its process does ALCESTE analyze each part of the text separately. This has the added value of managing the potential for human bias in the interpretation of the results: ALCESTE does not impose an interpretation according to which one part of the text is considered different from or similar to others. The researcher analyses quotations only once they have been identified as being either typical or atypical of a specific co-use of words. To put it another way, when the researcher analyses the quotations, s/he does so based on a report of how the language in each quotation is different from or similar to the language used in other quotations. Thus, the human coding is guided by an informative report, and human bias is controlled (Illia et al., 2012). The researcher receives a number of written or visual descriptions of the results. All of the elements listed below have been analyzed by the refinement process described above, until the meaning of each class is clear and the researcher gains a comprehensive understanding of how and why each cluster is distinct. This is the phase in which human interpretation takes place. In the *classification tree* or dendrogram, composed of macro-areas divided into stable clusters which correspond to lexical universes or “mental rooms” which the authors of the texts “enter” (Reinert, 1993), the distance trees visually represent the descending hierarchical classification. Each cluster is characterized by a *specific vocabulary*, composed of words which recur significantly more frequently inside the corpus, and by *a set of elementary context units* (*ECUs*), obtained through the analysis of punctuation and length (fundamental statistical units for the software). In each cluster, the frequency of words is only a secondary parameter, as frequency is relevant only when it reveals that there is a more frequent use of certain words in a specific part of the text, compared with another part of the text. This is indicated by the Chi^2^ for words in a class. The value of Chi^2^ is not absolute; it can only be computed within each class. All words listed in the report are relevant for interpretation, even considering that a high positive Chi^2^ indicates words that co-occur the most in a cluster (Illia et al., 2012).

## Results

We will describe the phases of the PAR cycle carried out in each school: planning, action, and reflection. The fourth phase (evaluation) is common to all three schools.

### Phase 1: Planning

#### The School in Rome

The teachers decided to form discussion groups about educational wastage that also included the students, followed by workshops and, finally, new discussion groups to discuss the workshops. During the first meeting, the teachers started by describing the negative characteristics of their pupils: unenthusiastic, passive, inept, uninterested, lazy, dull, distracted, flat. The students were also described in the following terms: they have no passion, they don’t care about politics, they have no interests; they don’t know how to communicate with each other; they are atomized, isolated, and divided; they don’t form a group; they don’t know how to communicate with teachers; they have no perception of their limits and resources; they have an inaccurate perception of (the institution’s) space and time; they are often compliant with the teachers, but not genuine; they do not follow the rules. It is not clear whether the characteristics the teachers discussed are subjective or context-bound. Only one teacher said: “it’s weird; they shouldn’t be bored at that age.” Teachers also formulated some interesting theories on the learning/teaching process, along the lines of the following: there is no learning without relationships, but it’s difficult here; if a student has specific learning difficulties, the family has to be involved, although they don’t usually cooperate; the clever ones have to leave, or they’ll waste away. The desire/goal of the teachers is to create an environment of trust and an alliance with the pupils. They listed the educational strategies employed thus far, to no avail, and the obstacles they met. Then they recounted a fragment of daily life in the classroom: “During an hour of substitution, someone in class throws a shoe in the air. The teacher asks who’s to blame for this violent and irresponsible gesture. The class joins forces to keep the secret.” In the follow-up discussion, the teachers said they fear the aggressiveness of their students, and for this reason they try to avoid any possible conflict. But one teacher said, “In my opinion, there is no education without conflict.” Then, they discussed the chance offered by the project to become equipped with a tool for observation and reflection in order to understand why the strategies used thus far do not work.

Later on, the teachers introduced the project to their students. The first meeting with the students followed, where students described themselves as suburban teenagers: as suburban teenagers, different from city center teens. As they said, in the suburbs, you become autonomous early and you start working early; if you leave the suburbs, you’re in trouble; the suburbs are freedom and the center is a cage, but it’s also true that the suburbs have no rules and are out of control, while the center is protected and follows the rules. They also illustrated theories explaining what is wrong with school, according to them: they want privacy, while adults ask questions and then judge and punish them. Because of this, they hide from the adults; sometimes they pretend to be interested, but it’s difficult to have a real dialogue between students and teachers, because the distance is too great. They discussed at length the fact that the teachers address the students with “tu” (informal language) while demanding to be addressed with “lei” (formal language). The teachers actively underline their institutional role. One of the teachers does so aggressively, sarcastically making fun of the student who is talking. The students’ desire to not be treated like children and to have more independence and be less controlled emerged. Then came the recounting of a moment of daily school life: “the other day, two girls beat each other up in the hallway. The teachers decided to add more controlling rules applying to all students”; and “all field trips have been cancelled for lack of trust in the students, who are all considered irresponsible.” During the discussion, the students said that the teachers forget what it means to be teenagers and don’t give second chances, and that it would be good to have teachers who really care about the students and give second chances.

The next meeting began in the midst of many organizational hindrances. The atmosphere was chaotic. Nevertheless, the teachers managed to discuss the issues raised by the students; some teachers critiqued the lack of strictness of present-day schooling, by now “reduced to a playroom.” Afterward, they started planning some experimental educational approaches, trying to answer the question: why is it that not even workshop-based pedagogy is successful with students?

Teachers then decided to come up with the following: a workshop on aggressiveness and rules of conduct during law classes; a workshop on the use of technology during English classes; and a lesson “that allows the students to take time to make mistakes and correct them” during maths classes.

#### The School in Bologna

During the first meeting, the teachers started by describing the negative characteristics of their pupils: they are difficult, suburban kids who are left out, disowned and different. They communicate through physical violence, speak a different language, and have no self-awareness, nor awareness of time and space, but are receptive, sometimes accepting the teachers’ assistance, letting themselves be guided. Additionally, they immediately pointed out the school’s strengths: open and democratic management, good relationships between colleagues, the school’s participation in supply requests, funding, and services, new teachers being seen as a resource, and the welcoming and protective environment. The school’s problems then followed: too many students drop out of school in the transition to high school, there is aggressiveness in the classroom, and some teachers are insulted. Afterward, they recounted an educational experience carried out the previous year: the teachers offered the students a workshop about Golding’s novel *Lord of the Flies*. The goal was to make the students understand the importance of rules. Their evaluations of the experiment emerged during the discussion: what caused the failure of the workshop were language issues, infantility, and the pace of learning differing between pupils.

They also recounted an experience with the students’ parents: the Women’s School, aimed at teaching Italian to immigrant mothers. During the discussion, many doubts emerged over the usefulness of involving the parents in this way.

Finally, they described another educational experience: a workshop on the topic of violence against women, based on newspaper readings. During the discussion, the teachers reported to have observed the presence of widespread misogyny and of an alarming aggressive tendency in the classroom.

The teachers proposed to investigate the causes behind the academic failure and school wastage of their pupils, distributing questionnaires or collecting information *post hoc*. But the methodological proposal, which had initially excited the teachers, was then abandoned. Later on, the teachers decided to introduce interdisciplinary workshops: a workshop about the telegraph, as it is the ancestor of modern immediate, long-distance communication; a workshop teaching how to plan a trip to a foreign country; and a workshop teaching how to build polygons using plastic straws.

#### The School in Naples

As already mentioned, in this Lower Secondary School, teachers have been cooperating with MdS for some time, offering workshops and accepting the help of its partners. Many times MdS has offered the school a reflective space for teachers, but opposition has been considerable. This school’s manifest problem is educational wastage in its many forms.

For the “Crossing Educational Boundaries” Project, we decided to observe some already-existing workshops, characterized by interactive methods and by the presence of a teacher, an expert on the subject, and an educator: a buoyancy workshop; a workshop about food composition; and a workshop about the food pyramid.

### Phase 2: Action

#### The School in Rome

Because of some new organizational issues, only one workshop was carried out: the one about aggression and rules of conduct, which used triggering images and created spaces of narration and discussion. The workshop was managed by an MdS pedagogue, but the teachers were present too. Some photos were projected: a picture of wolves baring their teeth but not attacking, and another of wolves playing. A debate arose among the students about the reasons behind animal and human aggression, about strategies to restrain it and control emotions, and about civilization and friendship.

#### The School in Bologna

All workshops were carried out and observed: the observer noticed that during the workshop involving learning the basics of the Morse alphabet, the students were divided into groups placed in different classrooms in order to try and communicate from a distance, and as a result many students were bored and some did not participate at all. During the workshop that involved a division into groups in order to use a computer to buy tickets, book accommodations, and plan museum visits and nights out, many conflicts erupted between single students or subgroups for the use of the computer, and some students did not participate at all. During the workshop consisting of building different polygons following the teacher’s instructions, using straws of different lengths, only one small group participated, while many students remained inactive.

#### The School in Naples

The first workshop began in a situation of confusion that caused a strong emotional reaction of discouragement in the teacher: “I can’t do it anymore! I wasn’t trained to educate, it’s not my job! I’m sick and tired of shouldering the education of other people’s children!” The chaos continued: some pupils stood up and wandered around, some exited the classroom, some strangers entered, one threw pencils, another isolated himself, some provoked others. According to the observer, the teacher became cynical with some students, mocking them for their physical features or for their mistakes. Then she managed to restore calm, and demanded loudly for the approval of the MdS partners: “Here, see, I shut them up.” In the second workshop, we found the same chaos, but here one of the students intervened to restore calm: “Shut up, we can’t work like this!” After a while, the chaos started again: some left the class, some entered, some joked around, some threw things, some ate, some used mobile phones, some ruined the others’ project. At this point, the teacher had a strong emotional reaction and started screaming in turn. The observer noted that when no adult in the classroom decides to stop the actions and talk to the students, chaos grows and the adults explode; when the educator intervenes by stopping the action and listening to the kids, calm is restored.

### Phase 3: Reflection

#### The School in Rome

During the final discussion, the teachers expressed their opinions: the workshop worked, but they don’t have time to do things like that, because they have to follow the syllabus. A single teacher says: “Actually, we could, we are free to choose.”

#### The School in Bologna

At the end of the workshops in this school, the teachers entrusted the reflection to their students. The observer commented that “they seem to struggle to talk about what they feel more than what they know.” Finally, the students said that working in groups is at the same time more tiring and more fun, and that they lacked the time to finish the workshop because the bell rang. A young girl said that today she felt good at school, she felt like she was at home.

After many organizational issues, the teachers suspended the research activities.

#### The School in Naples

A discussion meeting is held with teachers and students, in order to reflect on the workshops’ progress: the teachers decided to assign some students the task of cooperating and maintaining order, so that they are responsible. A third workshop started peacefully, then the usual chaos began. The teacher left the class often. This time, the educator took it upon herself to talk to the kids to make them reflect on what was happening and on a sense of the rules for working in a group.

A new discussion with the students followed, where they discussed their own self-representation: some kids said that they are seen as “monsters,” and that it’s true they are “monsters,” and that is why there is an observer.

During the final discussion, the teachers suggested to pupils that they keep diaries about what happens in class, to discuss them together later, but the kids say they don’t want to write negative things about their classmates. During the last group discussion, the students gave their evaluation of the workshop activities they took part in, using words like: nice, bad, tranquillity, serenity, happiness, relaxing. A girl adds: “I felt good, like at home.”

In this school as well, after some organizational issues, the research activities were temporarily suspended.

### Phase 4: Evaluation

Subsequently, MdS decided – together with the teachers – to entrust a team of external researchers with the textual analysis of the corpus composed of the observational reports. The initial results have been discussed with the teachers from all schools on the occasion of the International Convention “Crossing Educational Boundaries,” held in Naples in October 2017.

The corpus – composed of 18 observational reports – was pre-processed, as it underwent *disambiguation* and partial *lemmatization*. Each text was marked by three *illustrative variables*: city (Roma, Bologna, Napoli), type of school (Lower or Upper Secondary), and type of activity (discussion or workshop). The analyzed characteristics of the textual corpus are illustrated in Table 1.

**TABLE 1:** Textual corpus: quantitative characteristics.

Observation reports	18
Number of occurrences	28150
Distinct forms	5073
Minimum frequency of a word	6
Maximum frequency of a word	1099
Number of hapax (words that only appear once)	2874
Categorized Elementary Context Units (ECU)	511
Illustrative variables	3

**FIGURE 1:**
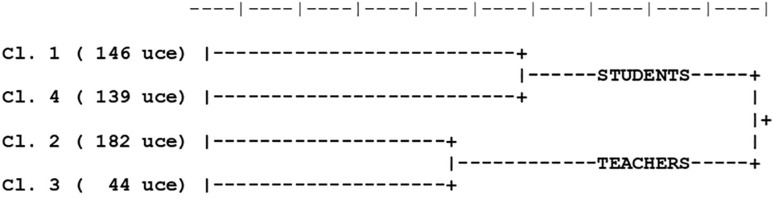
Descending hierarchical classification.

The analysis classified 511 *ECUs* out of the 732 detected (stability index 69.81%), divided in two macro-areas (A. Students; B. Teachers) and four stable clusters, as shown in the dendrogram of *ECU* classification (see Figure 1). For each cluster, the number and percentage of *ECUs* were indicated (see Table 2). Each cluster was assigned a thematic label on the basis of the specific vocabularies and of the most significant *ECUs*. Specific vocabularies and illustrative variables of clusters are illustrated in Table 2.^2^

**TABLE 2:** Clusters: specific vocabularies^a^ and illustrative variables.

**CLUSTER 1**		**CLUSTER 4**		**CLUSTER 2**		**CLUSTER 3**	
The students’ discourse on marginality		The students’ behavior between disorder and attentiveness		The teachers’ discourse on difficulties		The teachers’ behavior in the organization	

**Chi^2^**	**Words**	**Chi^2^**	**Words**	**Chi^2^**	**Words**	**Chi^2^**	**Words**

41.66	I	106.75	ANNA	39.29	professors	130.63	experimental
30.72	LUIGI	70.35	ROBERTO	28.76	students	118.75	(it) will be
31.59	we	67.4	GIUSEPPE	22.21	difficulties	107.86	meeting
29.43	because	54.08	educator X	20.75	school	97.92	experimentation
29.15	ANTONIO	53.21	professor	20.57	inside	96.62	activity
26.98	people	47.12	confusion	18.70	communication	76.56	course
26.72	(to) say	44.2	ROSA	15.75	relationship	75.03	meetings
26.55	maybe	38.64	begin	14.69	questionnaire	64.28	dates
24.33	our	37.86	classmates	13.28	research	64.28	feedback
23.97	FABRIZIO	31.88	(to) do	13.16	discourse	51.86	teaching
21.41	you (pl.)	28.57	(s/he) does	12.83	skills	45.52	thirty
20.32	SALVATORE	28.55	BENEDETTA	12.83	knowledge	45.52	involved
18.06	center	27.3	professor Y	10.98	(to) communicate	43.08	specific
18.06	friends	27.3	calm (adj)	10.98	awareness	42.79	conduct
17.74	(to) comment	27.3	calm (adj. pl)	10.98	curiosity	42.79	(they) will be
17.74	(I) feel	26.26	class	10.46	sense	42.79	agreed
16.33	STEFANO	25.79	(s/he) gets up	9.93	participation	39.24	fulfilment
16.33	(s/he) retorts	25.45	now	9.93	forms	39.24	(to) realise
15.62	freedom	24.52	minutes	9.41	group	35.59	emerged
15.28	(s/he) retells	24.52	(s/he) jokes	9.13	resources	35.03	hours
15.21	(we) are	24.10	while	9.13	outside	32.70	operators
15.18	(s/he) suggests	24.10	(s/he) re-exits	9.13	importance	32.70	observer
13.85	other	21.75	(to) enter	9.13	need	32.70	main
13.80	things	21.75	(s/he) goes out	9.13	understand	32.03	February
13.53	(s/he) wants	19.88	moment	9.13	ignite	32.03	educator
13.22	ENRICO	19.61	kids	9.13	alliance	29.99	March
12.62	truth	19.55	experiment	7.84	experiences	29.99	English
11.36	(s/he) exclaims	19.55	(to) exit	7.77	neighborhood	26.00	(to) define
11.36	in fact	19.10	(s/he) comes	7.77	ability	25.66	classes
11.36	your (pl.)	18.99	known	7.77	fundamental	22.58	workshops
11.36	really	18.99	desks	7.77	topics	22.58	(they) show
11.25	*dare del lei* ^∗^	17.60	desk	7.29	unanimously	21.45	project
10.87	language	16.93	after	7.29	scarce	21.24	day
10.87	friend	16.65	doing	7.29	spontaneity	17.69	way
10.08	UGO	16.61	to explain	7.29	comprehend	16.95	maths
10.08	PAOLO	16.25	hallway	7.29	process	16.95	impressions
10.08	GIANNI	16.25	calm (n.)	7.29	development	16.95	phase
10.08	convinced	16.25	ground	7.29	(to) organise	15.74	reflections
10.08	(you) can	16.25	(they) have to	7.29	questions	13.44	(to) observe
10.08	my	16.25	(to) stay	7.29	time	13.44	together
10.08	close	16.25	(to) sit down	7.1	possible	13.22	unit
10.08	(to be) available	14.36	focused	5.46	learning	12.93	April
9.24	says	14.18	(they) do	5.46	examine	12.93	offer
9.00	suburb	13.51	LINA	5.46	help	12.93	features
8.92	easy	13.51	(to) notice	5.46	computer	12.93	third
8.92	friendship	13.51	(they) get up	5.46	message	12.93	available
8.92	(we) pretend	13.51	hallways	5.46	world	12.93	observed
8.92	simply	13.51	(we) arrive	5.46	numbers	12.93	realization
8.58	reflects	13.51	(they) say	5.46	telegraph	12.93	participatory
8.58	image	13.51	(they) play			12.93	report
8.58	issue	13.51	to follow			12.00	observation
7.54	rather	13.51	(they) sit down			7.29	workshop
7.54	too much	12.27	(s/he) brings			7.12	agreement
7.54	control	12.27	(s/he) decides			7.12	aim
7.54	cage	12.15	often			6.71	first
7.54	majority	12.15	attention			6.32	content
7.54	(they) understand	11.83	as soon as				
7.54	concerns	11.09	hand				
7.48	good	10.79	FRANCESCO				
6.66	all	10.79	meanwhile				
6.54	proves	10.79	(they) start				
6.54	finally	10.79	throwing				
6.54	free	10.79	objects				
6.51	(we) have	10.79	arrogant				
6.51	malaise	10.79	calm				
6.39	concludes	10.79	vivacious				
6.20	(s/he) was	10.79	photo				
5.90	(they) have	10.79	disorder				
5.33	again/yet	9.82	(to) shout				
		9.67	circle				
		9.66	pause				
		8.08	PIERO				
		8.08	behind				
		8.08	now				
		8.08	violent				
		8.08	sheet				
		8.08	change				
		8.08	hands				
		7.11	teacher’s desk				
		7.11	(we) enter				
		7.01	classmate				
		6.44	silence				

**Illustrative variables:**		**Illustrative variables:**		**Illustrative variables:**		**Illustrative variables:**	

165.1	city ROME	428.13	city NAPLES	207.76	city BOLOGNA	39.58	activity DISCUSSION
165.1	school UPPER SECONDARY	146.35	school LOWER SECONDARY	148.85	activity DISCUSSION		
55.19	activity WORKSHOP	91.27	activity WORKSHOP	12.37	school LOWER SECONDARY		

*^*a*^In the translation from Italian to English it was often necessary to use more than one word to get across the original meaning. The words with a value of Chi^2^ up to about 5 are shown. ^∗^Non-translatable expression in English that indicates addressing the other in a formal way.*

#### I Macro-Area

The first macro-area identified by ALCESTE contains clusters 1 and 4, regarding, respectively, the words and actions of the students.

##### Cluster 1 – The students’ discourse on marginality (146 ECUs) (28.57%)

The specific vocabulary of Cluster 1 (see Table 2) contains many names of the students^3^ who participated in the discussion, and shows their self-representation as linked to the suburb they live in, with all its contradictions. The suburb – compared with the center – is considered a place of freedom, where it is easy to make friends, but also a cage with no institutional control. In school, they feel ill at ease, but this truth is hidden, because they pretend. Addressing teachers with “lei” (Italian formal language) creates too much distance in their relationship, and does not make them feel close. The associated illustrative variables show a significant correlation (see Table 2) with the school in Rome, where the students are older.

The content of the cluster becomes clearer by reading the specific *ECUs:*

1. “I have a friend of mine who used to live in the center and let’s say he was a normal person,” says Antonio, “now this friend of mine went to live in Prima Valle (suburban neighborhood) and yeah, I mean, he’s in trouble.”
2. “He recognized him because he has the double haircut, like the one I have …” says Stefano, tilting his head and showing off his nice haircut: “the double cut means you’re not from the center, that you’re an outsider. So, they recognized him and stabbed him!”
3. “In the suburbs, it’s easier for a kid to become autonomous on their own, of course! Because we grow up faster,” continues Fabrizio. “In these places, though, many kids find themselves forced to find personal autonomy far too soon.”
4. “It’s not like it’s easy to talk here at school,” Luigi tells us. “For example, I can’t stand having to address the teachers with ‘lei’: I think it would be much better to address them with ‘tu,’ because in this way I’d feel that they were closer and we could form a deep bond, of true friendship, or nearly so.”
5. “But me, in these kind of moments I don’t feel like saying anything, I just wanna stay quiet, by myself.”
6. “Sometimes I feel a bit bad, I feel down and I don’t feel like telling the reason why, or like saying what I feel, because maybe I don’t even really know.”
7. “From TV, from the internet, from friends, from teachers, even from our parents: they’re all there, telling us what to do, what to say and what to wear, sometimes it’s a good thing, but sometimes it’s bad. It’s good when people really care about us and can give us real directions, while still leaving us the freedom to choose.”

In Cluster 1’s most significant ECUs, some additional features of student self-representation emerge: exterior features, violent conflict between neighborhoods, and early autonomy outside the school. Students emphasize difficult communication with teachers. They emphasize this difficult communication with teachers, but also express the desire to be guided by adults who are authentically interested: “it’s good when people really care about us and can give us real directions, while still leaving us the freedom to choose.”

The illustrative variables associated with this cluster show that the discourse is, above all, from the older students and takes place during the workshops (presumably the narrative laboratory on aggressiveness).

##### Cluster 4 – The students’ behavior between disorder and attentiveness (139 ECUs) (27.20%)

Cluster 4’s specific vocabulary (see Table 2) contains numerous references to the workshop activities carried out in class (see illustrative variables associated). The names of students, teachers, and MdS educators are repeatedly mentioned; there are many verbs indicating activity and movement, and some words that explicitly refer to space and activities (such as to do, to begin, to enter, to re-exit, to go out, to come, to sit down, to get up, to arrive, to play, to bring, to throw). There are also many temporal indexes (like now, minutes, while, after, often); some words describe the atmosphere during the workshop, constantly alternating between chaos and involvement (like confusion, calm, disorder, attention, silence).

In Cluster 4’s most significant ECUs, we see clearly the constant attention swings of the class:

1. The kids stay enthusiastic about the experiments and explanations, which are not abstract but refer to their daily life, to concrete and practical examples. Massimo often leaves his desk, not to disrupt the class, but to see from up close what Barbara (workshop teacher) does. The kids are interested, they participate and ask questions, but the kid from before enters again and brings Luca a sandwich.
2. After 5 min they begin to pay attention. Then confusion starts again, so Barbara (workshop teacher) and Tonia (educator) raise their voices. No effect. So they change strategy, Barbara stays silent and Tonia tries to reason with the class about what is happening.
3. Now the (class’) teacher, who had left for 5 min, returns, and silence falls after her reprimand.
4. Massimo is interested, again he stands up and goes to see the experiment from up close, following Barbara (workshop teacher) while she walks among the desks. Tonia (educator) starts explaining from desk to desk, and as soon as she leaves Rosa’s table, she stands up. The group collapses, Rosa moves to a different desk, Enrico touches everything, Tina leaves the classroom.
5. During the distribution of the newspapers there is great confusion. Barbara (workshop teacher) talks, the kids stand up, tear the newspapers out of each other’s hands, the (class’) teacher tries to restore order by yelling and clapping her hands, while Tonia (educator) starts explaining the activity.
6. A great step forward was taken in such a short time. Even Mariarca raises her hand to be allowed to talk. Giuseppe enters the class again, I’m afraid he could bring chaos into this idyllic moment, but he leaves immediately, without creating confusion, because no one minds his presence, everyone stays seated in silence.

#### II Macro-Area

The second macro-area identified by the semantic-structural analysis contains clusters 2 and 3, regarding, respectively, the words and actions of the teachers.

##### Cluster 2 – The teachers’ discourse on difficulties (182 ECUs) (35.62%)

The specific vocabulary of Cluster 2 (see Table 2) contains the teachers’ words, uttered during the discussion groups (see also the associated illustrative variable). The teachers discussed their professional difficulties, related to the school and to the neighborhood. In particular, they focused on the students’ lack of skills, knowledge, awareness, curiosity and spontaneity, and on the need to understand and improve their relationship with them in order to better support their development and learning. Subsequently, the teachers discussed how they can participate in this research in its many possible forms, such as by creating questionnaires and working in groups; they discussed the time they would need, and the topics and experiences that might make sense for them and help them.

The content of the cluster becomes clearer by reading the specific *ECUs:*

1. Here, kids have a strong neighborhood identity. Their difficulties are due to the context of their origins, but not exclusively to that: the teachers unanimously agree on the matter.
2. The kids lack self-awareness, and awareness of their talents, their limits, their resources, and of their altered perception of space and time. The teachers continue working on an atmosphere of trust for the development of these skills, trying to build an alliance, or a pact, through alternative teaching activities, but to no avail.
3. There is a need for constant updates, because those who have been in the school system for a long time tend to consider some behaviors as natural and fail to embrace new ones. Some of the new teachers are a resource.
4. The professors think that a practice involving constant research and planning would be indispensable in order to be able to understand social changes.
5. The objective is to find a common language, improving communication with the class. All teachers make efforts every day to create a connection, but it is hard and the results are discouraging; nevertheless, they think it is possible.

In Cluster 2’s most significant ECUs, we see clearly some of the teachers’ ideas: in these schools, the kids have a strong sense of suburban identity, which causes some of their difficulties. There have been many tests of alternative teaching methods, aimed at creating an alliance with the students, but to no avail. Finally, there is a need for constant updates and research, especially for those who have been in the school system for a long time. Despite the discouragement, the teachers believe that change is possible.

##### Cluster 3 – The teachers’ behavior in the organization (44 ECUs) (8.61%)

Cluster 3 is the quantitatively least vast. Its specific vocabulary (see Table 2) contains references to the active organization of the research: dates, hours, setting (project, meeting, workshops, etc.), dynamic verbs (to bring about), verbs in the future tense (it will be, they will be), partners to involve (observer, educator), methods (experimentation, workshop observation), criteria for the choice of the classes to involve (to define), and accurate definition of the final phase (feedback, fulfilment, reflections, etc.).

The elementary context units specific to this cluster show commitment to the organization, but also some hindrances that stop the teachers from always being present during the activities they planned:

1. We organize the on-field work with the teachers present, and we co-plan the experimental teaching activities that will be offered to the different classes involved in the project.
2. The teachers seem available to start the experimental activities we agreed upon, and to establish the dates of the meetings over the telephone.
3. We will observe some classes held by the teacher, looking for answers about the main topics that emerged during the discussion.
4. Because of school obligations, the teachers S and P cannot take part in the meeting of February 14th.
5. Today the teachers are unavailable, because of the concurrence of another project.

#### Phase 5: New Planning

After the presentation of the results of this research to all the schools involved in this PAR, on the occasion of the aforementioned international convention, a small group of teachers discussed and planned, in cooperation with MdS, an intervention prototype: S.A.P.E.R.E.^4^ (Open Space for Planning And Educational Research). These prototypes are intended to defend resilience through collegiality and self-reflection: they are, in fact, groups of school teachers, educators and psychologists in the association who meet periodically for multi-vision meetings. The multi-vision group is a reflective methodology already successfully used by MdS partners (Parrello et al., 2019b). These prototypes are currently active in some schools in the Neapolitan suburbs, where the Association has made available its economic and professional resources.

## Discussion

This research is part of the many actions undertaken by MdS in order to contain the phenomenon of educational wastage in its multiple forms.

In deprived environments, school can be an important opportunity for development and social inclusion; instead, it often fails, as shown by the available data. In order to understand the reasons behind this partial failure, MdS deems it essential to actively involve the “main characters” of school life, equipping them with the resources necessary to produce potential change.

Following the theoretical perspectives that see educational wastage mainly as the effect of inadequate relationships, MdS has long since chosen the path of “taking care of the carers,” that is, to deal with the well-being of the adults responsible for educating the youth.

This project was born out of the experience gained in many Italian suburban schools: here – as it is also known from the literature – the difficulties of the students, and the high dispersion rate, make teachers feel ineffective or, worse, powerless, thus reducing their well-being at work and triggering a negative cycle damaging their educational performances.

T-PAR is a powerful form of professional development for teachers: it actively involves them, improving their awareness of themselves, of their emotions and actions, and of their power in that environment. However, T-PAR is not easily realized in schools: in the first place, because its multiple, open-ended goals would require the availability of the educational institution to accept modifications and unexpected changes. Secondly, because, in the beginning, it adds to the workload of the teachers without offering immediate advantages; and thirdly, because it can necessitate additional funding along the way, according to the paths undertaken. For these reasons, the first result to be emphasized here is therefore the realization of T-PAR in all its phases. In particular, the initial engagement phase was rather delicate: it was necessary to prevent the teachers from perceiving the research as if it were imposed by their superiors requesting their voluntary participation. The largest phase was planning in all schools, while the action phase has often encountered organizational obstacles, and the phase of reflection was always very short. But all phases – with their strengths and difficulties – were implemented in each school and documented by the observational reports.

In particular, in the school in Rome, the teachers decided to let the students talk from the start. This is an important, infrequent choice that involves students, and it allows the comparison of the discourses of adults and teenagers. Both ascribe the causes of academic failure to a difficult educational relationship and to lack of communication, but end up blaming each other for it; both complain about the strict limits of the institution. Both wish to find a way to communicate and change some of the school’s rules. A single teacher – a voice from *outside the box* – ultimately reminds the colleagues: “we are free to choose!” In the school in Bologna, the teachers reported impeccable functioning, and focused on the different features of workshop-based pedagogy, potentially able to create a serene climate in which learning is made easier. In the school in Naples, the well-established habit of being observed for MdS projects allowed the teachers to show their own strong emotional reactions, caused by the constant oscillation of the classes between chaos and attention. The discouraged teacher said, “I wasn’t trained to educate, it’s not my job!” Each school therefore seems to give emphasis to one aspect: the active involvement of students as co-researchers (Fielding, 2004), attention to alternative pedagogy (Esi, 2015), and/or the importance of the teacher’s emotional competence (Nouwen et al., 2016).

The semantic-structural analyses of the observational reports highlight additional important elements.

The students’ discourse (cluster 1) underlines the specificity of the environment. The suburb – compared with the center – is considered a place of freedom, a place which requires early autonomy, but is also a cage: some kids say that those who tried to “trespass” ended up in danger. In school they are not at ease, but they pretend. They emphasize difficult communication with teachers. They would like less controlling and more authentically interested teachers. In workshops, their behavior is inconsistent (cluster 4): they swing continuously from moments of attention and participation to moments of indifference and disruptive action; they are able to undertake the work, but they either do not want or cannot devote themselves to it with consistency. Perhaps even in the workshops realized in the three schools, where there is no asymmetry caused by the frontal lesson format, the issue of who controls and holds the power never disappears, and therefore ultimately carries weight. The teachers’ discourse (cluster 2) underlines the specificity of the environment, too: in the suburbs, it is difficult to teach and to apply theories, because it is hard to build a good educational relationship; here, according to the teachers, young people seem to have no awareness of their limits and possibilities, and they take a defensive stance, in order to oppose or to attack the adults. The observation of teachers’ behavior (cluster 3) shows how difficult it is to free themselves from the rigid and careless mechanism of the institution; organizational problems arise constantly, impeding full participation in the research. In all clusters, the lack of emotional vocabulary is striking. Teachers and students never mention emotions, while – as we have seen – the observers record multiple instances of behaviors definable as “acting out”: sarcastic displays, shouting, people leaving the classroom, disrupting actions from adults and adolescents alike.

These results suggest that, as assumed, the teachers used the professional support offered by MdS and their resources to analyze the problematic situations in which they are immersed. The general objective of the project was achieved, and hypotheses have been generated regarding the causes of educational wastage, identifying two potential areas of intervention: the improvement of teacher-student communication and of emotional competence. The second objective of the project was reached by a small group of teachers from various schools who, after the evaluation phase, started to plan actions to reduce the problem. Are they particularly “resilient” teachers? Remembering that resilience involves the capacity of an individual teacher to harness personal and contextual resources to navigate through educational challenges, we wonder if the project has supported their resilience and why it has failed to involve all the other teachers in the final phase (phase 5). In the context of the teaching profession, resilience may be conceptualized as a capacity, a process and also as an outcome: a dynamic process whereby characteristics of individual teachers and of their personal and professional contexts interact over time as teachers use specific strategies (Beltman, 2015). In particular, time and its management are fundamental resources: the most common professional challenge is lack of time due to heavy workloads and non-teaching duties such as paperwork or meetings (Castro et al., 2009; Beltman et al., 2011). The teachers denounced the “dual level” of the institutional mandate: the organization is separated from the exigencies of the educational relationship; and its scheduling and regulations fail to take into account the specificities of individuals and contexts, and are more and more oriented toward formality. Ultimately, school tends to hinder change. But, according to Enriquez (1972), the institution can become the place to cling onto in order to defend oneself from multiple anxieties.

“It follows that when the defensive component prevails, norms, role, schedules, technologies, etc., are used not as tools of work and development, but with the aim of never changing, of controlling everything and expecting everything, so to prevent the unexpected, or change, from creating anxiety. (…) Within organizations, everything is planned and thought-out (and happens consciously) as if the members and their relationships were moved solely by ideas and rational moments (…). However, in school there are also emotional, libidinal, affective factors (…). If the effect of these feelings over whether we support or obstruct each other in our cooperation and partnership with colleagues and pupils are not recognized, not only do we lose an occasion to work creatively, that is, to learn, but above all we contribute to keep the Institution unchangeable, namely we contribute to represent its defensive feature” (Blandino and Granieri, 1995, 95–96).

Ultimately, school is a place of paradoxes and contradictions. In order to avoid sacrificing any needs of the people and of the institution, pupils and teachers become masters of balancing acts: oscillating between participation and absence, cooperation and insubordination. However, this incessant oscillation can be seen in a positive perspective, as the attempt of both adults and young people to live within the school authentically, voicing their ambivalence. Of course, it is an attempt that requires energy and does not change the institution.

In conclusion, the results of this phase of the research contribute to the construction of a more precise picture of the situation of suburban schools, where the rates of educational wastage are high: the teachers who participated in our T-PAR are prepared on a theoretical level, are sensitive to the many problems of their pupils, and feel responsible as educators, but are also very distressed by the formal requests of the educational institution. In these conditions, they struggle to put their theoretical preparation to use, and fail to gain the trust of these young people and to give meaning to their job. What they get in return is a state of dissatisfaction that threatens their resilience.

Caring for horizontal and vertical relationships, the construction of an atmosphere of community within the school, and systematic reflection that gives space to the emotional and unconscious world should all be at the center of any project against educational wastage.

## Limits and Prospects

The specific limitations of this study involve the use of a single tool monitoring the intervention, that is observation, and the use of a single type of analysis. It would also be useful to complete this study by adding different analyses of the other schools’ research material.

In any case, these initial results propose school policies not limited to broadening the curriculum of suburban schools at risk of educational wastage: there is, additionally, the need to use further resources to improve the educational environment and to allow the teachers to systematically exercise professional self-reflection. To do so, it would be very useful to monitor the phase of research currently underway, which is the realization of intervention prototypes based on the use of reflective tools, in turn based on the model of the multi-vision group of MdS.

## Data Availability Statement

The datasets generated for this study are available upon request from the corresponding author.

## Ethics Statement

This study was carried out in accordance with the recommendations of the Declaration of Helsinki, and was approved by the Ethical Committee for Psychological Research (CERP) of the Department of Humanities of the University of Naples Federico II, prot. number 24/2019. The minors involved were only taking part in school activities that were included by the schools in their learning programs; the schools obtained an oral informed consent from the families at the beginning of the year. The CERP did not request further parental consent.

## Author Contributions

SP and CM designed the study and supervised the research. SP, II, and FC analyzed the data and wrote up the first draft of the manuscript. All authors interpreted the results, and approved the final version of the manuscript.

## Conflict of Interest

The authors declare that the research was conducted in the absence of any commercial or financial relationships that could be construed as a potential conflict of interest.

## Footnotes

1 According to Reinert (1990), creator of ALCESTE, a value of 50 is sufficient.

2 Since the collected data was in Italian, the resulting translation proved to be particularly difficult: it was necessary to come up with a partial approximation for some of the words where there was no exact correspondence with the English form.

3 All names were changed for privacy.

4 “Sapere” means *knowledge* in Italian.

## References

[B1] Ahola J. A. Lucas B. G. (1981). Participant observation in educational research: dynamics of role adjustment in a high school setting. *McGill J. Educ.* 16 76–90.

[B2] Alber S. R. Nelson J. S. (2002). Putting research in the collaborative hands of teachers and researchers: an alternative to traditional staff development. *Rural Special Educ. Q.* 21 24–32.

[B3] Arcidiacono C. Grimaldi D. Di Martino S. Procentese F. (2016). Participatory visual methods in the ‘Psychology loves Porta Capuana’ project. *Act. Res.* 14 376–392. 10.1177/1476750315626502

[B4] Arcidiacono C. Natale A. Carbone A. Procentese F. (2017). Participatory action research from an intercultural and critical perspective. *J. Prevent. Interv. Community* 45 44–56. 10.1080/10852352.2016.1197740 28084927

[B5] Aronson E. (2001). Integrating leadership styles and ethical perspectives. *Can. J. Admin. Sci.* 18 244–256. 10.1111/j.1936-4490.2001.tb00260.x

[B6] Avanzi L. Fraccaroli F. Castelli L. Marcionetti J. Crescentini A. Balducci C. (2018). How to mobilize social support against workload and burnout: the role of organizational identification. *Teach. Teach. Educ.* 69 154–167. 10.1016/j.tate.2017.10.001

[B7] Batini F. Bartolucci F. (eds) (2016). *Dispersione Scolastica: Ascoltare i Protagonisti Per Comprenderla E Prevenirla.* Milan: Franco Angeli.

[B8] Beltman S. (2015). “Teacher professional resilience: thriving not just surviving,” in *Learning to Teach in the Secondary School*, ed. Weatherby-Fell N., (Melbourne, VIC: Cambridge University Press), 20–38.

[B9] Beltman S. Mansfield C. Price A. (2011). Thriving not just surviving: a review of research on teacher resilience. *Educ. Res. Rev.* 6 185–207. 10.1016/j.edurev.2011.09.001

[B10] Betoret F. D. (2006). Stressors, self-efficacy, coping resources, and burnout among secondary school teachers in Spain. *Educ. Psychol.* 26 519–539. 10.1080/01443410500342492

[B11] Bizumic B. Reynolds K. J. Turner J. C. (2009). The role of the group in individual functioning: school identification and the psychological well-being of staff and students. *Appl. Psychol.* 58 171–192. 10.1111/j.1464-0597.2008.00387.x

[B12] Blandino G. Granieri B. (1995). *La Disponibilità Ad Apprendere: Dimensioni Emotive Nella Scuola E Formazione Degli Insegnanti.* Cortina: Milan.

[B13] Bobek B. L. (2002). Teacher resiliency: a key to career longevity. *Clear. House* 75 202–205. 10.1080/00098650209604932

[B14] Bondy S. (2001). Warming up to classroom research in a professional development school. *Contemp. Educ.* 72 6–8.

[B15] Brimer M. A. Pauli L. (1971). *Wastage in Education: A World Problem.* Paris: Unesco, IBE.

[B16] Brouwers A. Tomic W. Boluijt H. (2011). Job demands, job control, social support and self-efficacy beliefs as determinants of burnout among physical education teachers. *Eur. J. Psychol.* 7 17–39. 10.5964/ejop.v7i1.103

[B17] Brunetti G. J. (2006). Resilience under fire: perspectives on the work of experienced, inner city high school teachers in the United States. *Teach. Teach. Educ.* 22 812–825. 10.1016/j.tate.2006.04.027

[B18] Brydon-Miller M. Maguire P. (2009). Participatory action research: contributions to the development of practitioner inquiry in education. *Educ. Act. Res.* 17 79–93. 10.1080/09650790802667469

[B19] Capone V. Joshanloo M. Sang-Ah P. M. (2019). Burnout, depression, efficacy beliefs, and work-related variables among school teachers. *Int. J. Educ. Res.* 95 97–108. 10.1016/j.ijer.2019.02.001

[B20] Castro A. J. Kelly J. Shih M. (2009). Resilience strategies for new teachers in high-needs areas. *Teach. Teach. Educ.* 26 622–629. 10.1016/j.tate.2009.09.010

[B21] Cefai C. Cavioni V. (2014). “From neurasthenia to eudaimonia: teachers’ well-being and resilience,” in *Social and Emotional Education in Primary School: Integrating Theory and Research Into Practice*, eds Cefai C. Cavioni V., (New York, NY: Springer Science Business Media), 133–148. 10.1007/978-1-4614-8752-4_8

[B22] Chang M. L. (2009). An appraisal perspective of teacher burnout: examining the emotional work of teachers. *Educ. Psychol. Rev.* 21 193–218. 10.1007/s10648-009-9106-y

[B23] Corey S. (1953). *Action Research to Improve School Practices.* New York, NY: Columbia University.

[B24] David E. J. R. (ed.) (2014). *Internalized Oppression: The Psychology of Marginalized Groups.* New York, NY: Springer Publishing Co.

[B25] Day C. Gu Q. (2009). “Teacher emotions: well being and effectiveness,” in *Advances in Teacher Emotion Research: The Impact on Teachers’ Lives*, eds Schutz P. A. Zembylas M., (New York, NY: Springer), 15–31. 10.1007/978-1-4419-0564-2_2

[B26] Day C. Gu Q. (2014). *Resilient Teachers, Resilient Schools: Building and Sustaining Quality in Testing Times.* Oxon, UK: Routledge.

[B27] De Rosa B. Parrello S. Sommantico M. (2017). Ranimer l’espoir: L’intervention psycho-educative de Maestri di Strada. *Connexion* 107 181–196. 10.3917/cnx.107.0181

[B28] Enriquez E. (1972). Imaginaire social, refoulement et répression dans les organisations. *Connexions* 3 65–92.

[B29] Esi M. (2015). Adapting and integrating alternative didactics in the teaching-learning-assessment system in relation to the concept of ‘Disciplinary Field’. *Int. J. Soc. Educ. Innovat.* 3 7–12.

[B30] Fals-Borda O. (2001). “Participatory (action) research in social theory: origins and challenges,” in *Handbook of Action Research*, eds Reason P. Bradbury H., (Thousand Oaks, CA: Sage), 27–37.

[B31] Fielding M. (2004). Transformative approaches to student voice: theoretical underpinnings, recalcitrant realities. *Br. Educ. Res. J.* 30 295–311. 10.1080/0141192042000195236

[B32] Glanz G. (2003). *Action Research: An Educational Leader’s Guide to School Improvement*, 2nd Edn Massachusetts, MA: Christopher-Gordon Publishers, Inc.

[B33] Greenwood D. J. Whyte W. F. Harkavy I. (1993). Participatory action research as a process and as a goal. *Hum. Relat.* 46 175–192. 10.1177/001872679304600203

[B34] Gu Q. (2014). The role of relational resilience in teachers’ career-long commitment and effectiveness. *Teach. Teach.* 20 502–529. 10.1080/13540602.2014.937961

[B35] Hakanen J. J. Bakker A. B. Schaufeli W. B. (2006). Burnout and work engagement among teachers. *J. School Psychol.* 43 495–513. 10.1016/j.jsp.2005.11.001

[B36] Halbesleben J. R. B. (2006). Sources of social support and urnout: a meta-analytic test of the conservation of resources model. *J. Appl. Psychol.* 91 1134–1145. 10.1037/0021-9010.91.5.1134 16953774

[B37] Hall B. (1992). From margins to center? The development and purpose of participatory research. *Am. Sociol.* 23 15–28. 10.1007/bf02691928

[B38] Hastings R. P. Bham M. S. (2003). The relationship between student behaviour patterns and teacher burnout. *Educ. Psychol. Rev.* 21 193–218. 10.1177/0143034303024001905

[B39] Henderson N. Milstein M. M. (2003). *Resiliency in Schools: Making It Happen for Students and Educators.* Thousand Oaks, CA: Corwin press.

[B40] Hooks B. (1984). *Feminism: A Movement to End Sexual Oppression. S. Kemp J. Squires, Feminisms.* Oxford: Oxford University Press.

[B41] Hooks B. (1994). *Teaching to Transgress: Education as a Practice of Freedom.* NewYork, NY: Routledge

[B42] Howard S. Johnson B. (2004). Resilient teachers: resisting stress and burnout. *Soc. Psychol. Educ.* 7 399–420. 10.1007/s11218-004-0975-0

[B43] Illia L. Sompar K. Bauer W. (2012). Applying co-occurrence text analysis with ALCESTE to studies of impression management. *Br. J. Manag.* 25 352–372. 10.1111/j.1467-8551.2012.00842.x

[B44] Istat. (2015). *Noi Italia: 100 Statistiche per Capire Il Paese In Cui Viviamo.* Rome: ISTAT.

[B45] Jacobs S. (2016). The use of participatory action research within education-benefits to stakeholders. *World J. Educ.* 6 48–55. 10.5430/wje.v6n3p48

[B46] Johnson G. M. (1997). Resilient at-risk students in the inner city. *McGill J. Educ.* 32 35–49. 10528454

[B47] Johnson M. J. Button K. (2000). Connecting graduate education in language arts with teaching contexts: the power of action research. *Eng. Educ.* 32 107–126.

[B48] Jones S. J. (2003). Complex subjectivities: class, ethnicity, and race in women’s narratives of upward mobility. *J. Soc. Issues* 59 803–820. 10.1046/j.0022-4537.2003.00091.x

[B49] Kawulich B. (2012). “Collecting data through observation,” in *Doing Social Research: A Global Context*, eds Wagner C. Kawulich B. Garner M., (New York, NY: McGraw Hill), 150–160.

[B50] Kidd S. A. Kral M. J. (2005). Practicing participatory action research. *J. Couns. Psychol.* 52 187–195. 10.1037/0022-0167.52.2.187

[B51] Kyriacou C. (2001). Teacher stress: directions for future research. *Educ. Rev.* 53 27–35. 10.1080/00131910120033628

[B52] Lavy S. Ayuob W. (2019). Teachers’ sense of meaning associations with teacher performance and graduates’ resilience: a study of schools serving students of low socio-economic status. *Front. Psychol.* 10:823. 10.3389/fpsyg.2019.00823 31057458 PMC6482213

[B53] Le Cornu R. (2013). Building early career teacher resilience: the role of relationships. *Aust. J. Teach. Educ.* 38 1–16. 10.14221/ajte.2013v38n4.4

[B54] Lewin K. (1948). *Resolving Social Conflicts.* New York, NY: Harper.

[B55] Lott B. (2012). The social psychology of class and classism. *Am. Psychol.* 67 650–658. 10.1037/a0029369 23163450

[B56] Marcosa J. Miguela E. Tillemab H. (2009). Teacher reflection on action: what is said (in research) and what is done (in teaching). *Reflect. Pract.* 10 191–204. 10.1080/14623940902786206

[B57] McMahon L. Farnfield S. (2010). *Too Close in or Too Far Out: Learning to Hold the Role of Observer.* Oxford: Oxford University Press.

[B58] McMillan J. H. Reed D. F. (1994). At-risk students and resiliency: factors contributing to academic success. *Clear. House* 67 137–140. 10.1080/00098655.1994.9956043

[B59] McTaggart R. (1997). “Guiding principles for participatory action research” in *Participatory action research: International contexts and consequences*, ed McTaggart R. (Albany, NY: SUNY Press), 25–43.

[B60] Ministry of Education, University and Research [MIUR], (2014). *Dossier “Early school leaving”.* Available at: https://www.tuttoscuola.com/dossier/ecco-il-dossier-dispersione-di-tuttoscuola-scaricalo/ (accessed October 01, 2019).

[B61] Molloy Elreda L. Jennings P. A. DeMauro A. A. Mischenko P. P. Brown J. L. (2019). Protective effects of interpersonal mindfulness for teachers’ emotional supportiveness in the classroom. *Mindfulness* 10 537–546. 10.1007/s12671-018-0996-y

[B62] Morales M. P. E. (2016). Participatory action research (PAR) cum action research (AR) in teacher professional development: a literature review. *Int. J. Res. Educ. Sci.* 2 156–165.

[B63] Nouwen W. Van Praag L. Van Caudenberg R. Clycq N. Timmerman C. (2016). *School-Based Prevention and Intervention Measures and Alternative Learning Approaches to Reduce Early School Leaving.* Belgium: Centre for Migration and Intercultural Studies, University of Antwerp.

[B64] Ocasio W. Joseph J. (2005). An attention-based theory of strategy formulation: linking micro- and macroperspectives in strategy processes. *Adv. Strateg. Manag.* 22 39–61. 10.1016/S0742-3322(05)22002-8

[B65] OCSE (2015). *Indagine OCSE PISA 2015: I Risultati Degli Studenti Italiani in Scienze, Matematica e Lettura.* Available at: http://www.invalsi.it/invalsi/ri/pisa2015/doc/rapporto_PISA_2015.pdf

[B66] OCSE-PISA (2012). *Programme for International Student Assessment.* Available at: http://www.invalsi.it/invalsi/ri/pisa2012.php?page=pisa2012_it_07

[B67] Parrello S. (2018). “Growing up in the suburbs: stories of adolescents at risk and of their “maestri di strada”,” in *Idiographic Approach to Health*, eds De Luca Picione R. Nedergaard J. Freda M. F. Salvatore S., (Charlotte, NC: Age Publishing), 161–176.

[B68] Parrello S. Ambrosetti A. Iorio I. Castelli L. (2019a). School burnout, relational and organizational factors. *Front. Psychol*. 10:1695. 10.3389/fpsyg.2019.01695 31428010 PMC6688533

[B69] Parrello S. Iorio I. De Rosa B. Sommantico M. (2019b). Socio-educational work in at-risk contexts and professional reflexivity: the multi-vision group of “Maestri di Strada”. *Soc. Work Educ.* 1–16. 10.1080/02615479.2019.1651260

[B70] Pellerone M. Ramaci T. Miccichè S. (2018). Identity, family, relationships among groups and socioeducational disadvantage as factors of school failure: a cross-sectional study in a group of junior high school students of the sicilian hinterland. *World Futures* 74 321–342. 10.1080/02604027.2018.1492293

[B71] Perone E. (2006). *Una Dispersione Al Plurale: Storie Di Vita Di Giovani Che Abbandonano La Scuola Nella Tarda Modernità.* Milan: Franco Angeli.

[B72] Pine G. J. (2009). *Teacher Action Research: Building Knowledge Democracies.* Thousand Oaks, CA: Sage.

[B73] Reinert M. (1990). Alceste, une méthodologie d’analyse des données textuelles et une application: aurélia de gérard de nerval. *Bull. Méthodol. Sociol.* 26 24–54. 10.1177/075910639002600103

[B74] Reinert M. (1993). Les mondes lexicaux et leur logique à travers l’analyse statistique d’un corpus de récits de cauchemars. *Lang. Soc.* 66 5–39. 10.3406/lsoc.1993.2632

[B75] Sammons P. Day C. Kington A. Gu Q. Stobart G. Smees R. (2007). Exploring variations in teachers’ work, lives and their effects on pupils: key findings and implications from a longitudinal mixed-method study. *Br. Educ. Res. J.* 33 681–701. 10.1080/01411920701582264

[B76] Savin-Baden M. Wimpenny K. (2007). Exploring and implementing participatory action research. *J. Geogr. High. Educ.* 31 331–343. 10.1080/03098260601065136

[B77] Schön D. A. (1983). *The Reflective Practitioner: How Professional Think in Action.* New York, NY: Basic Books.

[B78] Skaalvik E. M. Skaalvik S. (2009). Does school context matter? Relations with teacher burnout and job satisfaction. *Teach. Teach. Educ.* 25 518–524. 10.1016/j.tate.2008.12.006

[B79] Soini T. Pyhältö K. Pietarinen J. (2010). Pedagogical well-being: reflecting learning and well-being in teachers’ work. *Teach. Teach.* 16 735–751. 10.1080/13540602.2010.517690

[B80] Solomon P. R. (1989). “Dropping out of academics: black youth and the sports subculture in a cross-national perspective,” in *Drop-Outs from School. Issues, Dilemmas and Solutions*, eds Weils L. Farrar E. Petrie H. G., (Albany, NY: New York Press), 79–93.

[B81] Sommantico M. Donizzetti A. R. De Rosa B. Parrello S. Osorio Guzmán M. (2015). Testing gender invariance of Italian version of aggression questionnaire (AQ) by Buss and Perry. *Psicologia della Salute* 3 111–125. 10.3280/PDS2015-003006

[B82] Stanford B. H. (2001). Reflections of resilient, persevering urban teachers. *Teach. Educ. Q.* 28 75–87.

[B83] Stapleton S. R. (2018). Teacher participatory action research (TPAR): a methodological framework for political teacher research. *Act. Res.* 0 1–18. 10.1177/1476750317751033

[B84] Steffens N. K. Haslam S. A. Schuh S. C. Jetten J. Van Dick R. (2017). A meta-analytic review of social identification and health in organizational contexts. *Pers. Soc. Psychol. Rev.* 21 303–335. 10.1177/1088868316656701 27388779

[B85] Susman G. I. Evered R. D. (1978). An assessment of the scientific merits of action research. *Admin. Sci. Q.* 23 582–603. 10.2307/2392581

[B86] UNESCO (1972). *Étude Statistique Sur les Déperditions Scolaires.* Available at: http://unesdoc.unesco.org/images/0000/000022/002227FB.pdf

[B87] Ungar M. (ed.) (2012). *The Social Ecology of Resilience: A Handbook of Theory and Practice.* New York, NY: Springer.

[B88] Van Roy K. Vanheule S. Inslegers R. (2015). Research on balint groups: a literature review. *Patient Educ. Couns.* 98 685–694. 10.1016/j.pec.2015.01.014 25681874

[B89] Zurlo M. C. Pes D. Capasso R. (2016). Personality characteristics, job stressors, and job satisfaction: main and interaction effects on psychological and physical health conditions of italian school teachers. *Psychol. Rep.* 119 27–38. 10.1177/0033294116656818 27381410

